# Glucagon increases energy expenditure independently of brown adipose tissue activation in humans

**DOI:** 10.1111/dom.12585

**Published:** 2015-11-20

**Authors:** V. Salem, C. Izzi‐Engbeaya, C. Coello, D. B. Thomas, E. S. Chambers, A. N. Comninos, A. Buckley, Z. Win, A. Al‐Nahhas, E. A. Rabiner, R. N. Gunn, H. Budge, M. E. Symonds, S. R. Bloom, T. M. Tan, W. S. Dhillo

**Affiliations:** ^1^ Section of Investigative MedicineHammersmith Hospital, Imperial College LondonLondonUK; ^2^ Imanova Centre for Imaging Sciences, Hammersmith HospitalLondonUK; ^3^ Department of ComputingImperial College LondonLondonUK; ^4^ Department of RadiologyImperial College NHS Healthcare TrustLondonUK; ^5^ Division of Brain SciencesImperial College LondonLondonUK; ^6^ Early Life Research Group, Academic Division of Child Health, Obsterics and Gynaecology, School of Medicine, Queen's Medical Centre, University HospitalThe University of NottinghamNottinghamUK; ^7^ Centre for Neuroimaging SciencesKing's CollegeLondonUK

**Keywords:** energy expenditure, ^18^F‐FDG PET/CT, glucagon, human brown adipose tissue, thermal imaging

## Abstract

**Aims:**

To investigate, for a given energy expenditure (EE) rise, the differential effects of glucagon infusion and cold exposure on brown adipose tissue (BAT) activation in humans.

**Methods:**

Indirect calorimetry and supraclavicular thermography was performed in 11 healthy male volunteers before and after: cold exposure; glucagon infusion (at 23 °C); and vehicle infusion (at 23 °C). All volunteers underwent ^18^F‐fluorodeoxyglucose (^18^F‐FDG) positron emission tomography (PET)/CT scanning with cold exposure. Subjects with cold‐induced BAT activation on ^18^F‐FDG PET/CT (n = 8) underwent a randomly allocated second ^18^F‐FDG PET/CT scan (at 23 °C), either with glucagon infusion (n = 4) or vehicle infusion (n = 4).

**Results:**

We observed that EE increased by 14% after cold exposure and by 15% after glucagon infusion (50 ng/kg/min; p < 0.05 vs control for both). Cold exposure produced an increase in neck temperature (+0.44 °C; p < 0.001 vs control), but glucagon infusion did not alter neck temperature. In subjects with a cold‐induced increase in the metabolic activity of supraclavicular BAT on ^18^F‐FDG PET/CT, a significant rise in the metabolic activity of BAT after glucagon infusion was not detected. Cold exposure increased sympathetic activation, as measured by circulating norepinephrine levels, but glucagon infusion did not.

**Conclusions:**

Glucagon increases EE by a similar magnitude compared with cold activation, but independently of BAT thermogenesis. This finding is of importance for the development of safe treatments for obesity through upregulation of EE.

## Introduction

Obesity results from an excess of caloric intake relative to energy expenditure (EE). There are now 600 million adults who are obese [body mass index (BMI) >30 kg/m^2^] worldwide. Obesity is causally linked to the development of type 2 diabetes, cardiovascular disease, some cancers 1 and early mortality. Lifestyle strategies for weight loss are based on calorie restriction, alongside increasing EE via exercise, but these efforts are difficult to sustain in the long term. Currently licensed drugs, generally aimed at either reducing intestinal fat absorption (such as intestinal lipase inhibitors) or inhibiting appetite (such as phentermine), have limited weight loss efficacy on their own. Anti‐obesity agents which elevate EE by stimulating the sympathetic nervous system have produced dangerous side effects, particularly cardiovascular morbidity 2, 3, 4. There is a great unmet need for drugs that can safely inhibit appetite and increase EE.

One possible solution is a pharmacological agent that acts as a co‐agonist of both glucagon‐like peptide‐1 (GLP‐1) and glucagon receptors, harnessing the anorectic and glucose‐lowering effects of the former with increased EE via the latter 5, 6. Glucagon infusion acutely increases EE in humans 7, 8, but the mechanism by which this occurs is not known. Rodents can increase their EE via activation of brown adipose tissue (BAT), which consumes fuel for thermogenesis using uncoupling protein‐1 (UCP‐1), and do so in response to both cold exposure and caloric excess 9. UCP‐1‐positive BAT deposits that can be activated by a variety of stimuli, most potently cold exposure, have been recently described in adult humans, particularly in the supraclavicular neck region 10, 11, 12.

Isolated brown fat cells from rats have been shown to respond thermogenically to glucagon 13 and glucagon administration to rodents increases BAT mass and activity 14. Glucagon knockout mice have reduced thermogenic responses to cold exposure and pharmacological adrenergic stimulation, which is restored by glucagon replacement 15, although the participation of BAT in the thermogenic response to glucagon administration is not firmly established 16; therefore, glucagon may increase EE in humans via activation of BAT, but studies investigating this are lacking.

We carried out a series of experiments to investigate, for the first time, whether the increased EE resulting from acute glucagon administration was mediated by BAT activation in adult humans.

## Materials and Methods

### Subjects

A total of 11 healthy men (mean age 26.1 years, range 20.8–39.8 years, mean BMI 22.5 kg/m^2^, range 20.5–25.2 kg/m^2^) were recruited through advertisement and assessed as healthy during a screening visit with a medical history, routine blood tests and ECG. Exclusion criteria were smoking, substance abuse, eating disorders, regular medication and medical or psychiatric illness. The study was approved by the London Central Ethics and Research Committee (13/LO/0925), registered with ClinicalTrials.gov (NCT01935791) and performed in accordance with the Declaration of Helsinki. Informed consent was obtained from subjects before enrolment in the study.

### Study Visits

Cold exposure and glucagon infusion have both been shown to acutely elevate EE in humans but have never been directly compared in the same cohort. We performed this comparison in 11 healthy young men. Each subject underwent measurement of resting metabolic rate (RMR) using an indirect calorimeter (Gas Exchange Monitor; GEM Nutrition, Daresbury, UK) at the start and end of three separate interventions: cold exposure, glucagon infusion in a warm room and vehicle infusion in a warm room. During these visits, we also used infra‐red thermography of the neck, which has recently been reported to be a reliable method of measuring BAT thermogenesis 17, 18. The calorimetry/thermal imaging visits were randomly assigned and are summarized below:

- Calorimetry and thermal imaging wearing a cooling vest (Polar Products®, Stow, OH, USA) 19 with a 55‐min intravenous (i.v.) vehicle (Gelofusine®; B. Braun, Sheffield, UK) infusion (cold visit).
- Calorimetry and thermal imaging in a warm room (ambient temperature 22–25 °C) with a 55‐min i.v. vehicle infusion (control, vehicle visit).
- Calorimetry and thermal imaging in a warm room (ambient temperature 22–25 °C) with a 55‐min i.v. glucagon infusion at 50 ng/kg/min (Novo Nordisk, Gatwick, UK; glucagon visit).

^18^F‐fluorodeoxyglucose (^18^F‐FDG) positron emission tomography (PET)/CT scanning is the most widely accepted method of identifying metabolically active BAT. All 11 subjects attended an initial ‘cold’ ^18^F‐FDG PET/CT scan study visit (described below), to ascertain if they were BAT‐positive or BAT‐negative.

Eight of the 11 subjects were confirmed as having supraclavicular BAT deposits on their initial ^18^F‐FDG PET/CT scan, based on standardized uptake value (SUV) characteristics previously defined 20. This BAT‐positive cohort then participated in a 5th study day, when a second ^18^F‐FDG PET/CT scan was performed, randomized to either: ^18^F‐FDG PET/CT with a 55‐min glucagon infusion at 50 ng/kg/min at 23 °C (n = 4) or ^18^F‐FDG PET/CT with a 55‐min vehicle infusion at 23 °C (n = 4).

**Figure 1:**
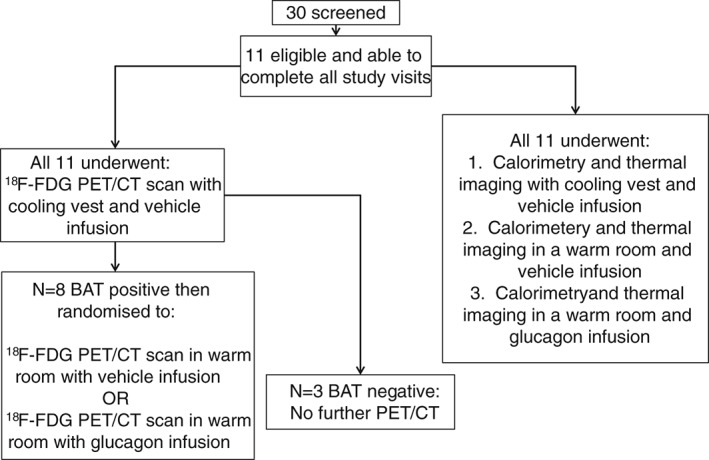
Study visit summary. BAT, brown adipose tissue; ^18^F‐FDG, ^18^F‐fluorodeoxyglucose; PET, positron emission tomography.

The study design is summarized in Figure 1 and timelines are detailed in Figure S1A–C. Subjects fasted and drank only water from 22:00 hours the night before each study visit. They ate the same meals the day before every study visit, abstained from alcohol, caffeine and avoided strenuous exercise for 24 h before each visit. Each visit was separated by at least 3 days. On arrival, peripheral venous cannulae were inserted in both forearms (one for infusion and one for blood sampling). Volunteers were blinded to the contents of the i.v. infusions. RMR was measured at the start and end of each thermal imaging visit using an indirect calorimeter (Gas Exchange Monitor; GEM Nutrition). At the start of each study visit the calorimeter was calibrated with ‘zero’ (0.00% O_2_ and 0.00% CO_2_) and ‘span’ (20.00% O_2_ and 1.00% CO_2_) gases (BOC, Guildford, UK). Human glucagon was purchased from Novo Nordisk (UK); 1 mg was diluted into 50 ml Gelofusine and the flow rate through the Alaris® syringe pump was adjusted to a maintenance i.v. delivery rate of 50 ng/kg/min. This dose was chosen based on previous work in our department to produce an acute rise in EE comparable with other studies examining the effects of cold exposure on EE and BAT activity 21. To ensure plateau plasma levels were quickly attained when glucagon was delivered, infusions were ramped at 4× maintenance rate (MR) for the first 5 min, 2 × MR for the next 5 min and 1 × MR for the remainder of the 55‐min infusion. The Polar Products cooling vest used in this experiment has been previously confirmed to successfully induce cold‐activated BAT without needing to change the ambient temperature of the experimental room 19. The waistcoat‐shaped vest was worn by the participants directly over a thin cotton hospital gown. Cold water (maintained at 8 °C by attaching to a temperature‐controlled tank) was continuously pumped through the vest. The subjects felt cold but none of them reported (or were observed to be) shivering (this was checked every 15 min).

### Thermal Imaging Protocol

Volunteers wore a pair of light cotton trousers only. On the cold visit day the cooling jacket was wrapped around the volunteer's torso leaving the supraclavicular regions uncovered. After a 30‐min acclimation period, baseline thermal images were recorded for 10 min using a Flir T440bx infrared camera (Flir Systems, West Malling, UK) mounted on a tripod placed 1 m from the subject. The subjects always sat fully upright in the same chair, with head and arm rests maintained in the same positions. Volunteers were asked to remain as still as possible, with their shoulders held unrotated against the back of the chair to minimize movement within the image frames during thermal recordings. Immediately after the baseline thermal imaging session, subjects were placed under the hood of the calorimeter for the baseline RMR recording (for 15 min).

At t = −10 min the infusion (vehicle or glucagon) was started and, on the cold visit day, cold water was pumped through the vest to begin the cooling process. The infusions were stopped at t = 45 min and the cold vest maintained for a further 45 min. Thermal imaging recordings were taken at baseline (Run A), just after the start of the intervention (Run B), and the final run (Run C) coincided with the end of infusion or cold vest exposure. Immediately after, a final RMR measurement with the hood calorimeter was also recorded (Figure S1A, B). Blood samples were taken at t = −55, −10, 0, 10, 15, 30, 45 and 60 min (see below).

### ^18^F‐FDG PET/CT Protocol

On arrival, peripheral venous cannulae were inserted in both forearms. Volunteers wore light cotton trousers. After 30 min acclimation, at t = −75 min, the cooling vest was put on and cold water circulated through for 60 min before the start of the scan. The vest was later draped over the volunteers' legs whilst in the scanner. On the second visit (vehicle or glucagon infusion) no cooling step was taken. At t = −10 min the volunteers entered the scanner and the infusion was started (ramped for the first 10 min, as previously described). Before the PET scan, an anatomical CT scan of neck and thorax was performed. At t = 0 min, 180 MBq of ^18^F‐FDG was injected i.v. and a 60‐min dynamic emission scan was performed, with an axial field of view from mandible to mid‐thorax (Figure S1C). Blood samples were taken at t = −55, −10, 0, 1, 3, 5, 10, 15, 30, 50 and 60 min.

### Blood Sampling

Samples for analysis of plasma glucagon were collected in lithium heparin tubes containing 0.15 ml aprotinin (Trasylol; Bayer Schering Pharma, Berlin, Germany) and immediately underwent centrifugation, after which plasma was promptly separated and stored at −20 °C until analysis using a radioimmunoassay 22. Glucagon and glucose was measured at all time points and catecholamines (plasma epinephrine and norepinephrine) were measured on the start and end samples, by the Department of Chemical Pathology, Imperial College Healthcare National Health Service Trust. Human fibroblast growth factor (FGF)‐21 levels in the start and end samples were measured using an enzyme‐linked immunosorbent assay (Merck Millipore, Darmstadt, Germany).

### Image Analysis

**Figure 2:**
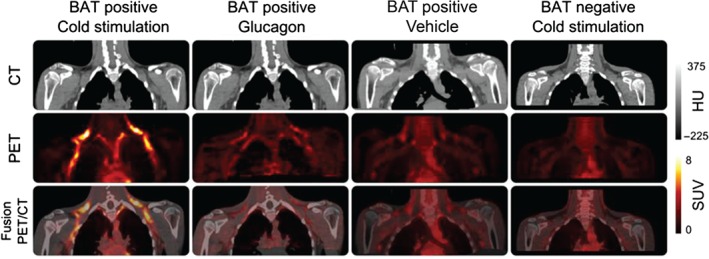
Example positron emission tomography (PET)/CT images. Example CT (top row), PET (middle row) and fusion PET/CT (bottom row) images. Column 1 is a subject who displays brown adipose tissue (BAT) activation during cold stimulation (one of the BAT positive cohort). Column 2 is of the same subject as Column 1, but when he received glucagon infusion on his second scan. Column 3 is a different subject who also displayed BAT activation on cold exposure, but showing his scan in response to vehicle in a warm room. Column 4 shows images from a volunteer who did not demonstrate BAT activation during cold stimulation (one of the BAT negative cohort). PET images are average activity between 30 and 60 min normalized to injected dose and body weight. HU, Hounsfield units; SUV, standardized uptake value.

For each subject, the second visit CT scan was non‐linearly registered and warped to the first visit CT scan using a multi‐resolution free‐form deformation technique, measuring the mutual information as a similarity measure between CTs. The three‐dimensional warp field estimate was subsequently applied to the dynamic PET of the second visit. The adipose tissue region of interest (ROI) was defined using tissue density on CT (−150 < Hounsfield Units < −5). A BAT ROI was then defined as adipose tissue with an SUV of ≥2 on the cold scan. Time–activity curves for BAT were generated by applying the ROIs to the dynamic PET data from both visits. Arterial plasma radioactivity was estimated using a whole‐blood time–activity curve derived from an aorta ROI scaled by the plasma to blood ratio derived from the discrete blood samples. Using the graphical Patlak model, estimates of the metabolic rate of FDG were calculated. Finally, the metabolic rate of glucose [MR(gluc)] was calculated by dividing the metabolic rate of FDG by the lump constant (lump constant = 1.14) in adipose tissue, 23 and multiplying by the concentration of glucose in the plasma. Representative images are shown in Figure 2.

**Figure 3:**
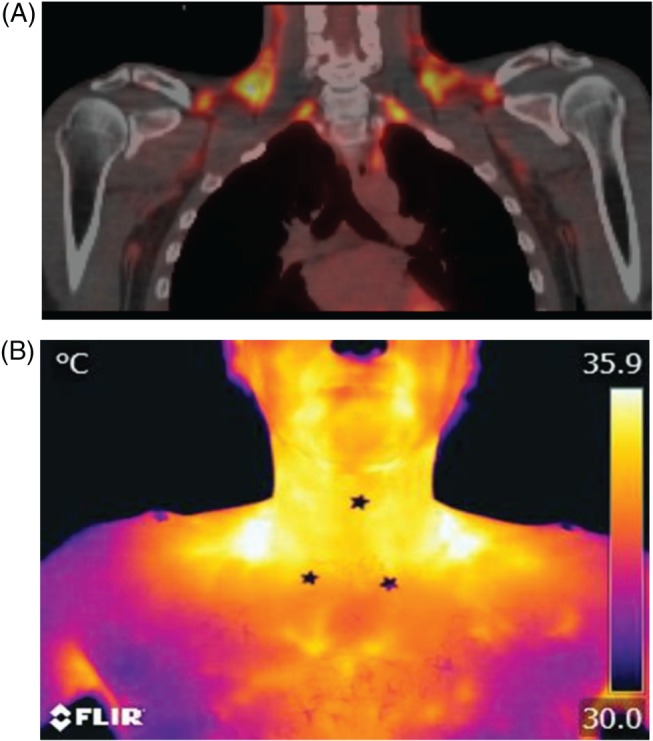
Examples of positron emission tomography (PET)/CT and thermal images from the same subject at the end of a cooling vest visit. (A) Fused ^1^ ^8^F‐fluorodeoxyglucose (^18^F‐FDG) PET/CT image (yellow areas represent increased metabolic activity) of a brown adipose tissue (BAT)‐positive subject during cold stimulation. (B) Thermal image taken at the end of the same cold exposure from the same subject. The stars on the thermal image were used to define the anatomical landmarks used, which defined the region of interest for temperature analysis.

Thermal recordings taken with Flir Tools Plus software (Flir Systems) were saved as stills every 30 s. Supraclavicular ROIs, defined by a triangle limited by the acromioclavicular joint, cricoid prominence and the sternoclavicular joint, were drawn on the first image using Thermacam Pro software (Flir Systems) and repositioned on all subsequent stills from that run. Data from each ROI were exported into Excel, where each cell recorded a camera pixel temperature reading, and a macro was created to calculate the mean temperature of the top 10% hottest pixels. Visual comparison of which pixels within the thermal image ROI were being included in the temperature analysis (by highlighting those falling into the top 10% of pixel temperatures) confirmed in all cases that the hottest areas in the supraclavicular ROI formed a contiguous cluster located on skin overlying a depot of BAT (Figure 3). As a comparator (control) region, we also measured the temperature changes in an ROI defined over the deltoid region for every thermal image. The deltoid ROI (an area known to be devoid of underlying BAT) was defined as a right‐angled triangle extending from the acromioclavicular joint to the lateral extremity of the deltoid.

### Statistical Analysis

Based on previous studies of the hormonal and cold exposure effects on human BAT activity 24, 25, we calculated that, for an equivalent rise in EE, a sample size of 4 per group (glucagon versus control) would have 95% power to detect a significant difference between the effects of cold exposure and glucagon using ^18^F‐FDG PET/CT in confirmed BAT‐positive volunteers. Because of safety considerations, as PET/CT scans expose healthy patients in this study to ionising radiation, we limited this study to males only and to the smallest possible sample size to answer our initial question about the differential effects of cold exposure and glucagon administration on human BAT activity for a given rise in EE.

All data were analysed using graphpad prism 6 software (GraphPad Software, Inc., San Diego, CA, USA). Results are presented as means ± standard error of the mean. One‐way repeated measures anova with Tukey's test was used to compare differences between the three intervention groups. A p value < 0.05 was considered to indicate statistical significance.

## Results

### Cold Exposure and Glucagon Infusion Produce a Similar Acute Rise in Energy Expenditure in Adult Humans

**Figure 4:**
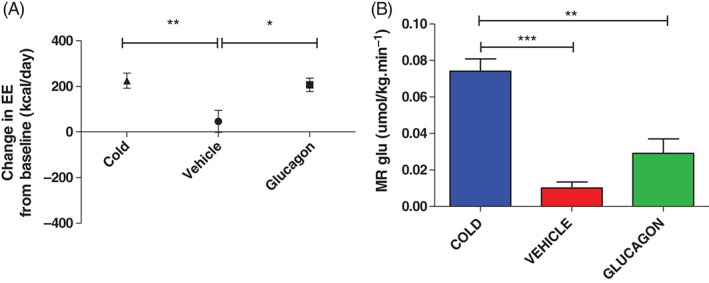
Effects of cold exposure, vehicle infusion and glucagon infusion in a warm room on (A) energy expenditure (EE) and (B) metabolic rate of glucose uptake [MR(gluc)] in brown adipose tissue (BAT) during ^18^F‐fluorodeoxyglucose (^18^F‐FDG) positron emission tomography (PET)/CT. RMR was measured with an indirect calorimeter at the start and end of each intervention and the mean change from baseline are shown in (A). Baseline resting EEs were as follows 1279 ± 59 kcal/day (cold), 1353 ± 52 kcal/day (vehicle) and 1315 ± 39 kcal/day (glucagon), and were not significantly different from one another. Data are presented for all 11 volunteers (separated into BAT‐positive and BAT‐negative cohorts in Figure S4). (B) Shows MR(gluc) in BAT during ^18^F‐FDG PET/CT determined under the same experimental conditions: cold exposure (blue bar), vehicle only (ambient temperature 23 °C) (red bar) and glucagon infusion (green bar). Results are expressed as means ± standard error of the mean, **p < 0.01, ***p < 0.001.

Cold exposure with a cooling vest and 50 ng/kg/min glucagon infusion cause a similar rise in RMR (∼14 and 15% from baseline, respectively) compared with vehicle: 193.0 ± 27.2 kcal/day with cold, 41.1 ± 70 kcal/day with vehicle and 230.8 ± 30.1 kcal/day with glucagon (Figure 4A). The baseline respiratory quotients (respiratory quotient: *V* co_2_/*V* o_2_) in each group were not significantly different from one another [0.797 ± 0.016 (cold); 0.788 ± 0.017 (vehicle); and 0.765 ± 0.021 (glucagon)] but rose significantly with glucagon infusion [change from baseline in respiratory quotient by end of intervention: −0.012 ± 0.009 (cold), −0.003 ± 0.001 (vehicle), and +0.055 ± 0.016 (glucagon, p < 0.01)], which was indicative of the expected rise in rate of carbohydrate oxidation with glucagon, in line with its glucose‐liberating effects and hence the relative substrate availabilities of glucose versus free fatty acid, and consistent with our previous data 7, 8. Plasma levels of glucagon rose only during the glucagon infusion, which is shown in Figure S2 alongside plasma glucose and insulin levels. Previous studies have suggested that glucagon stimulates FGF‐21 secretion in humans 26 and that FGF‐21 stimulates BAT. We found a non‐significant rise in circulating FGF‐21 levels in our cohort in response to glucagon infusion, also detailed in Figure S2.

Circulating norepinephrine levels rose significantly with cold exposure but not with vehicle or glucagon infusion [cold 2.33 ± 0.14 to 2.84 ± 0.33 mmol/L (p < 0.05), vehicle 1.95 ± 0.18 to 1.74 ± 0.18 mmol/l and glucagon infusion 2.19 ± 0.18 to 2.02 ± 0.26 mmol/l]. Circulating epinephrine levels did not change (data not shown) in any group. Heart rate and blood pressure data are presented in Figure S3. There was a small rise in heart rate with glucagon infusion, consistent with its well‐described chronotropic effects. In comparison, cold‐induced peripheral vasoconstriction and hence a rise in peripheral vascular resistance may account for the measured rise in mean arterial pressure with cold exposure. In general, these findings support the conclusion that for a similar rise in RMR between glucagon infusion and cold exposure, glucagon infusion induces less sympathetic outflow.

### Cold Exposure but not Glucagon Infusion Activates Human BAT Despite a Similar Rise in Energy Expenditure

We tested the hypothesis that the increased EE resulting from acute glucagon administration to adult humans was mediated by BAT activation using both ^18^F‐FDG PET/CT and thermal imaging.

#### *^18^F‐FDG PET/CT*

In the eight subjects confirmed as having active supraclavicular BAT deposits, BAT mass ranged from 4.8 to 92.9 g, mean 36.7 ± 11.1 g. In our cohort of BAT‐positive volunteers, the MR(gluc) in BAT was significantly higher with cold exposure than both vehicle and glucagon infusion. Although there was a small trend in BAT activation after glucagon infusion, there was no statistical difference in BAT MR(gluc) between vehicle and glucagon infusion (Figure 4B): mean BAT [MR(gluc)] with cold exposure 0.074 ± 0.007 µmol/kg/min, vehicle 0.010 ± 0.003 µmol/kg/min and with glucagon 0.029 ± 0.008 µmol/kg/min. In line with this, a breakdown of the EE rise between the BAT‐positive and BAT‐negative groups (Figure S4) showed that the rise in EE induced by glucagon in BAT‐negative subjects was not less than that in the BAT‐positive group, confirming that glucagon has an effect on RMR independent of BAT activity.

#### *Thermal Imaging*

**Figure 5:**
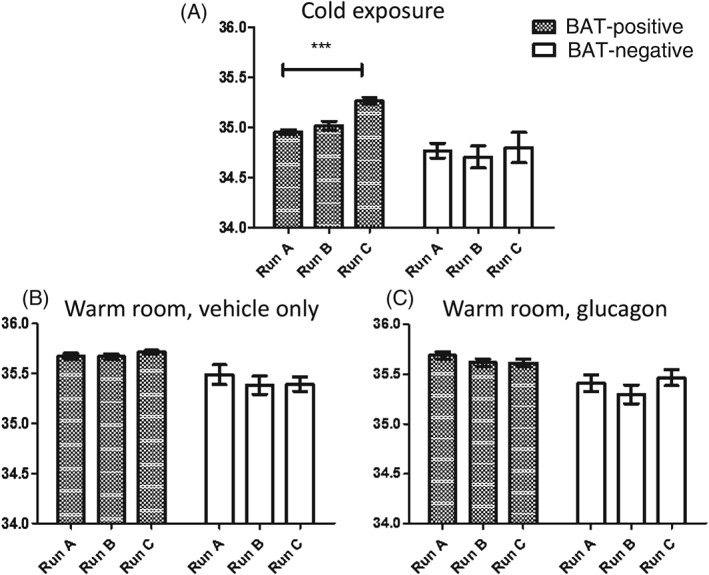
(A–C) Average supraclavicular region of interest temperature (°C). Results are shown for baseline (Run A), mid‐intervention (Run B) and end intervention (Run C) for each visit type. Each run represents a 10‐min thermal recording, with stills extracted every 30 s and an average reading of the upper 10% pixels calculated. These were then averaged across the cohort of brown adipose tissue (BAT)‐positive [as defined on positron emission tomography (PET)/CT; n = 8, hatched bars] and BAT‐negative (n = 3, white bars). (A) Response to cold exposure (cooling vest). (B) Response to vehicle infusion in a warm room. (C) Response to glucagon infusion in a warm room. Results are expressed as means ± standard error of the mean, ***p < 0.001.

All 11 subjects (eight BAT‐positive and three BAT‐negative, as defined by their ^18^F‐FDG PET/CT characteristics on cold exposure, described above) also underwent thermography of their neck during the three study visits when calorimetry was performed (Figures 1 and S1A, B). A rise in supraclavicular skin surface temperature has previously been shown to indicate underlying BAT thermogenic activity 17, 27. In the BAT‐positive group of eight, there was a significant temperature rise in the neck with cold exposure, with a mean increase in temperature between Run A (baseline) and Run C (end exposure) of 0.44 ± 0.08 °C. In contrast, there was no change in supraclavicular neck temperature with cold exposure in the BAT‐negative group (Figure 5A). These findings confirm that thermal imaging was able to identify independently the same individuals who had cold‐induced BAT activation as shown by ^18^F‐FDG‐PET/CT. In both the BAT‐positive and BAT‐negative groups, thermal imaging showed no change in neck temperature when subjects received either vehicle (Figure 5B) or glucagon infusion (Figure 5C). As a comparator (control) region, we plotted the temperature changes in an ROI defined over the deltoid region (an area known to be devoid of underlying BAT) for all of the thermal images (Figure S5). All of the conditions (vehicle in a warm room, glucagon in a warm room and cold exposure) were associated with a fall in the skin temperature over the deltoid, as the experimental protocol required them to sit with the torso/shoulders exposed; however, the fall in deltoid temperature in the vehicle and glucagon visits was very similar (−1.05 and −1.09 °C respectively) whereas the fall in temperature in response to the cold exposure was much greater (−1.90 °C). These data suggest that the rise in BAT‐positive supraclavicular temperature with cold exposure is even more pronounced (relative to the drop in skin temperature elsewhere).

Taken together, these data show that cold exposure and glucagon infusion cause a similar rise in EE, but only cold exposure is associated with significant thermogenic activation of BAT in the acute setting.

## Discussion

This study is the first to measure the effects of glucagon infusion on BAT activity in humans. We found that glucagon and cold exposure induced a similar acute rise in EE in humans, but in this setting only cold exposure, and not glucagon, induced the activity of supraclavicular BAT in adult humans measured using either ^18^F‐FDG‐PET/CT or thermal imaging. We also found that, although cold exposure was capable of increasing sympathetic activation (as determined by steady‐state norepinephrine levels), glucagon infusion did not significantly alter circulating norepinephrine levels. These findings support the conclusion that, whilst acute cold exposure and acute glucagon infusion produce a very similar rise in RMR, the effector mechanisms through which EE is elevated are different. These findings are of importance to drug development based on glucagon receptor agonism, as the dose of glucagon used in the present study increased EE significantly without sympathetic system activation, thus potentially avoiding the deleterious effects on the cardiovascular system that have been encountered with some weight‐loss medications.

In 2009, metabolically active BAT in adult humans was described in detail 10, 11. This has led to a resurgence of interest in the utility of BAT as a tantalizing target for safely increasing EE to aid weight loss. Enhanced BAT activity may also induce favourable effects on glucose homeostasis and circulating lipids 28. Exposure to cold temperatures is the most potent stimulator of BAT metabolic activity, which has been correlated with the associated rise in EE that occurs in the cold [although not so in this particular study (Figure S6)]. More recently, the concept of repeated cold exposure as a means of recruiting or upregulating BAT for therapeutic purposes has also been reported 29, although the feasibility of such an approach in a clinical setting remains to be seen. The effector mechanism for cold‐induced thermogenesis is the sympathetic nervous system, which richly innervates BAT, via the β3 adrenoceptor. In line with this, patients with catecholamine‐secreting tumours have evidence of increased BAT activity, which abates once the tumour is removed, and thyrotoxic patients show a similar upregulation of BAT activity, as thyroid hormone potentiates β‐adrenergic signalling 25, 30. Conversely, a study very similar to the present study described the effects of non‐selective β‐adrenergic stimulation by isoprenaline in healthy young men on BAT activity. Vosselman et al. 24 found that, although isoprenaline infusion increased EE to the same extent as cold exposure, it did not significantly activate BAT, indicating that other tissues are responsible for generalized β‐adrenergic thermogenesis. Indeed, early preclinical trials of β3 agonists as anti‐obesity agents did not progress because of poor efficacy 31. A more recent study, using newer and more highly specific β3 agonists (licensed for other conditions), however, suggests that pharmacological manipulation of BAT remains a viable option. Cypess et al. 32 described significant BAT activation in response to a single dose of the selective β agonist mirabegron, associated with a 200 kcal/day rise in EE, although this was not reported against the degree of measured cold‐induced BAT activity in the cohort. More recently, Broeders et al. 33 described the effect of bile acids on human BAT activation, reporting a small but significant rise in BAT SUV_max_, from 1.0 ± 0.4 to 1.6 ± 0.4, compared with 7.2 ± 5.4 in acute cold exposure. They therefore concluded that the maximum achievable BAT activation in these subjects far exceeded the effects of chenodeoxycholic acid, but that more potent bile acid BAT activators may have traction as anti‐obesity drugs.

^18^F‐FDG‐PET/CT, the imaging method chosen for all of these BAT studies, is the currently accepted ‘gold standard’ method for measuring BAT volume and activity. In rodent studies, thermographic measurement of BAT activity has been validated against biochemical markers of BAT thermogenesis, such as UCP‐1 upregulation 34. In human studies, Jang et al. 17 reported that a temperature difference between the neck and sternum of 0.9 °C had a positive predictive value of 85% for the presence of supraclavicular BAT, Symonds et al. 18 recorded rises of 0.5 °C in supraclavicular temperature in children after mild cold exposure, and Boon et al. 27 reported a 0.4 °C cold‐induced supraclavicular skin temperature rise, which positively correlated with ^18^F‐FDG‐PET/CT quantification of underlying BAT. In the present study all eight subjects who had BAT activation after cold exposure as determined by ^18^F‐FDG‐PET/CT also showed a rise in neck temperature, as determined by thermal imaging during cold exposure, although the correlation between PET‐quantified BAT mass and cold‐induced neck temperature rise was not significant in the present cohort (data not shown). We also observed that the rise in neck temperature occurs within the first 10 min after cold exposure and remains elevated for the duration of the exposure. By contrast, the subjects with an absence of cold‐activated BAT activity on their PET/CT scan displayed no change or a fall in neck temperature under cold conditions. In all cases, both saline and glucagon infusions (in a warm room) produced no rise in neck temperature on thermal imaging, supporting the conclusion that glucagon produces at best only a very modest effect on BAT activity in this experimental setting.

To further check the validity of our thermal findings, we manually overlaid all thermal images on the corresponding PET/CT images. In all eight BAT‐positive subjects, the 10% hottest pixels from the thermal image (that were used for our data analysis) consistently formed a contiguous cluster overlying the largest depot of BAT defined on that subject's corresponding PET/CT. Furthermore, when we analysed the temperature change in the pixels that overlaid BAT on the corresponding PET/CT, the same temperature rise was found as produced by the (blinded) whole ROI analysis, confirming that analysis of the entire ROI accurately reflected skin temperature changes directly overlying a confirmed BAT depot. In the three BAT‐negative subjects, the 10% hottest pixels were more sparsely distributed across the entire ROI. These findings support the use of thermal imaging to investigate BAT activity by directly measuring the output of the uncoupling process, namely heat. Development of such novel methods for quantifying and measuring the function of human BAT is important, as the restoration or upregulation of BAT activity in humans may provide a non‐surgical means of treating obesity. Indeed, although ^18^F‐FDG PET/CT is still generally regarded as the gold standard for measuring BAT activity, the technique is expensive, exposes research participants to ionizing radiation and may not be the best measure of BAT metabolism as circulating glucose is used as a substrate for BAT thermogenesis only after internal fatty acid stores and then circulating free fatty acids have been used. Ouellet et al. have reported on the utility of using other PET tracers, such as ^11^C‐acetate (to determine tissue oxidative activity) and the fatty acid ^18^F‐fluoro‐thiaheptadecanoic acid (^18^FTHA) to measure non‐esterified fatty acid uptake in human BAT 35. Others have focused on other methods of imaging without the need for exposure to ionising radiation, such as fat‐fraction MRI to quantify BAT mass coupled with functional MRI to investigate BAT activation, measuring blood flow as a marker of thermogenesis 29.

The present work has important therapeutic implications. Modern estimates suggest that BAT activity could account for 5% of the basal metabolic rate in humans 36, although its maximum inducible potential remains unknown. Two previous studies have reported on the addition of chronic glucagon receptor agonism to the antidiabetic properties of GLP‐1 receptor agonism in diet‐induced obese mice 5, 6. Co‐agonism of glucagon and GLP‐1 receptors induced superior body weight loss and improvements in glucose homeostasis than the GLP‐1 receptor agonist alone, and caused a significant rise in EE. These findings have led to intense interest in the concept of balanced co‐agonism of both the glucagon and GLP‐1 receptors to achieve superior weight loss as a result of the additional EE effects of glucagon receptor activation. We confirmed the applicability of this finding to humans, showing that glucagon acutely increases EE in humans 7, and the present study builds on our understanding of the mechanism by which it does so.

The dose of glucagon used in the present experiment was based on our previous knowledge of the dose required to significantly increase EE in humans. We have previously shown that a dose of 50 ng/kg/min (i.e. 14 pmol/kg/min) i.v. glucagon over 45 min increased resting EE significantly: by a mean of 146.99 kcal/day in healthy overweight individuals 7. This dose, administered over 55 min in the present study (notably on a leaner and younger cohort) produced a 206.8 ± 29.6 kcal/day rise in EE. In another recent publication, we showed that a dose of 2.8 pmol/kg/min i.v. glucagon over 130 min (i.e. a fifth of the dose used in the present study but over twice the time) produced a non‐significant rise in EE of 66.8 kcal/day in overweight volunteers (n = 13) 8. The plateau plasma levels measured in this experiment following glucagon infusion (∼300 pM) are also consistent with those previous reports, although higher than those typically expected when fasting (∼50 pM); however, this would not be unexpected in the setting of a pharmacological (weight‐loss) agent containing a glucagon agonist element. Notably, we have chosen to present our EE data in units of kcal/day, thereby extrapolating our acute measurement of EE rise with glucagon to the 24‐h period. Whilst there is strong animal data that chronic GLP‐1 receptor and glucagon‐receptor co‐agonism produces long‐lasting weight loss effects and increased EE, the effects of chronic glucagon receptor agonism in humans are not known. Thus, whilst a 24‐h extrapolated value for the EE effects of glucagon infusion may not necessarily be reflective of what would happen with prolonged glucagon agonism over that period (or longer), it gives a sense of the potential therapeutic effect of this strategy.

We powered the present study to detect a significant difference between the effects of cold exposure and glucagon using ^18^F‐FDG PET/CT in confirmed BAT‐positive volunteers. With the data presented in this study, we can confidently state that, for the same rise in EE, glucagon has less effect on BAT than cold, although it is not possible to exclude a small effect of glucagon on human BAT activity with this study. Furthermore, although BAT activity was measured on PET/CT during the 55 min i.v. infusion of glucagon when plasma levels had reached a plateau, cold exposure was initiated 1 h before the scan, which may also have militated against detecting an effect of glucagon on BAT activity. To reduce the contribution of cold‐activated BAT on the placebo or glucagon study days, we maintained the scanner room temperature at 23 °C throughout the study. Indeed, it would be interesting to further investigate the interplay between constitutive BAT activity (as occurs in mild cold stress) and its responsiveness to glucagon, which was not answerable with this particular study. As we wished to limit radiation exposure to women of childbearing potential, the data presented are for young, healthy males only. Nevertheless, our findings imply the existence of an as‐yet unidentified, and potentially targetable, glucagon‐driven pathway to enhanced EE. Other mechanisms by which glucagon may be exerting its thermic effects have still to be explored in humans in greater detail. Futile substrate cycling induced by non‐physiological glucagon receptor activation provides a means to increasing the metabolic rate through stimulation of energy‐consuming cyclical metabolic pathways, with no net change in product formation, in the liver and other tissues. Tracer studies have indicated a significant effect on glucose cycling in response to glucagon infusion, an effect that occurs predominantly at low insulin levels and is abolished with high‐dose insulin infusion. The contribution of this to whole‐body EE remains to be adequately explored 37, 38. Glucagon also has well described positive inotropic and chronotropic effects on the myocardium, although the small contribution of the heart to total RMR could not account for the rise in EE that we have measured after glucagon infusion 39. Finally, we have not excluded a small element of BAT activation in humans in response to glucagon administration, and the effects of chronic glucagon receptor activation on BAT mass and function in humans also remain unknown. In line with animal data, this may include upregulation of classical BAT deposits versus the possibility that chronic glucagon receptor agonism may promote WAT transdifferentiation to a more brown‐like phenotype. The present study has highlighted these important areas for future research into the hormonal upregulation of human EE as a safe therapeutic option for obesity.

## Conflict of Interest

The authors have nothing to disclose.

V.S., C.I.‐E., E.R., R.G., S.R.B., T.T. and W.S.D. designed the experiment; V. S., C. I.‐E., C. C., A. C., and A. B. carried out the experiments; V. S., C. I.‐E., C. C., D. T., Z. W., N. A. N., E. R. and R. G. performed the image analysis and performed the statistical analyses. V.S., C. I.‐E. and W. S. D. wrote the manuscript. All authors contributed to editing the final manuscript and had final approval of the submitted article. W.S.D. is the guarantor of this work, had full access to all the data, and takes full responsibility for the integrity of data and the accuracy of data analysis.

## Supporting information


**Figure S1.** (A) Timeline for calorimetry and thermal imaging vist, cooling vest with vehicle infusion. (B) Timeline for calorimetry and thermal imaging vist, warm room with vehicle or glucagon infusion. (C) Timeline for PET/CT visit.

**Figure S2.** (A) Plasma glucagon levels. (B) Plasma glucose levels. (C) Plasma insulin levels. (D) Change in fibroblast growth factor‐21 levels between start and end of intervention.

**Figure S3.** (A) Mean pulse rate (measured every 15 min) of all 11 subjects during exposure to the cooling vest (blue bar), vehicle infusion in a warm room (red bar) and glucagon infusion in a warm room (green bar). (B) Change in mean aterial pressure [estimated from diastolic (DBP) and systolic (SBP) measurements as DBP + 1/3(SBP − DBP)] between the start and end of the experimental exposures of cooling vest, vehicle and glucagon infusions.

**Figure S4.** Effects of cold exposure and glucagon infusion in a warm room on energy expenditure separated into brown adipose tissue (BAT)‐positive (n = 8) and BAT‐negative (n = 3) groups.

**Figure S5.** Average deltoid (control) region of interest temperature (°C) (A–C) and neck (brown adipose tissue‐positive) region of interest temperature (°C) (D–F).

**Figure S6.** Scatter plots for brown adipose tissue (BAT) activity and energy expenditure (EE) rise. (A) BAT metabolic activity [MR(gluc)] (µmol/kg/min) averaged across the cold PET/CT scan against the percentage rise in cold‐induced EE (as measured on the calorimetry day, under the same conditions of cold exposure). (B) BAT MR(gluc) against glucagon‐induced rise in EE.
