# Supplementary material for: Glucagon increases energy expenditure independently of brown adipose tissue activation in humans
Source: Diabetes Obes Metab. 2015 Nov 20;18(1):72–81. doi: 10.1111/dom.12585 (PMC4710848; doi:10.1111/dom.12585)
Supplement: Supplementary file 1 — Figure S1. (A) Timeline for calorimetry and thermal imaging vist, cooling vest with vehicle infusion. (B) Timeline for calorimetry and thermal imaging vist, warm room with vehicle or glucagon infusion. (C) Timeline for PET/CT visit. [file dom0018-0072-sd1.docx]

**Supplemental Figures S1a to S1c: Study visit timelines (related to Main Figure 1, study visit summary).**

11 male subjects attended for 4 or 5 study visits (Main Figure 1) and underwent (in random order)

- Thermal imaging and indirect calorimetry wearing a cooling vest with a 55-minute vehicle infusion (cold visit, Supplemental Figure S1a)
- Thermal imaging indirect calorimetry in a warm room (ambient temperature 22–25°C) with a 55-minute vehicle infusion (control visit, Supplemental Figure S1b)
- Thermal imaging indirect calorimetry in a warm room with a 55-minute glucagon infusion at 50 ng/kg/min (glucagon visit, Supplemental Figure S1b)

All 11 subjects also underwent an ^18^F-FDG PET/CT scan (Supplemental Figure S1c). The first PET/CT scan was always wearing the cooling vest with a 55-minute vehicle infusion. If this scan revealed cold-induced BAT activity, then the second PET/CT visit occurred, randomised to be either:

- - ^18^F-FDG PET/CT in a warm room (ambient temperature 23°C) with a 55-minute glucagon infusion at 50 ng/kg/min OR
  - ^18^F-FDG PET/CT in a warm room with a 55-minute vehicle infusion

**
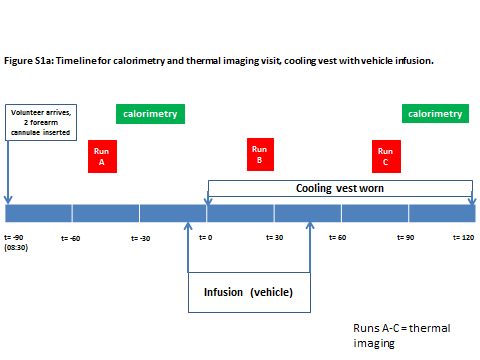
**

**
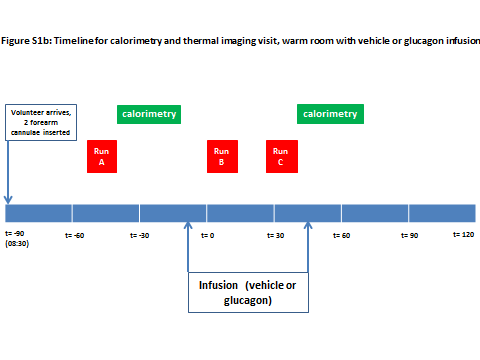
**

**
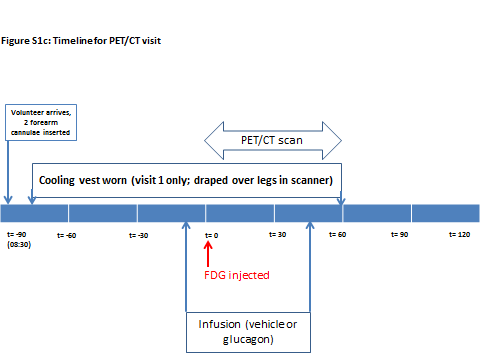
**
