# Supplementary material for: Glucagon increases energy expenditure independently of brown adipose tissue activation in humans
Source: Diabetes Obes Metab. 2015 Nov 20;18(1):72–81. doi: 10.1111/dom.12585 (PMC4710848; doi:10.1111/dom.12585)
Supplement: Supplementary file 2 — Figure S2. (A) Plasma glucagon levels. (B) Plasma glucose levels. (C) Plasma insulin levels. (D) Change in fibroblast growth factor‐21 levels between start and end of intervention. [file dom0018-0072-sd2.docx]

**Supplemental Figure S2: Plasma glucagon levels (S2a), glucose (S2b), insulin (S2c) and FGF-21 (S2d) levels.** Plasma levels are plotted by study intervention for all 11 subjects during exposure to the cooling vest (blue legends), vehicle infusion in a warm room (red legends) and glucagon infusion in a warm room (green legends). All data points shown as mean ± SEM.

**S2a: Plasma levels of glucagon** peaked at 370 ± 87 pmol/L at 40 minutes following the start of the infusion and promptly fell back to baseline 10 minutes after discontinuation. Plasma levels of glucagon during the vehicle infusion and cooling vest protocols remained below 20 pmol/L and there were no differences between plasma (endogenous) glucagon levels measured in the cold exposure or (warm) vehicle visits.

End infusion

Start infusion

**S2d: Change in plasma FGF-21 levels between start (t=0) and end (t=60) intervention.** There was a non-significant rise of 238 ± 197 pg/ml in circulating FGF-21 following glucagon infusion.
