# Supplementary material for: Glucagon increases energy expenditure independently of brown adipose tissue activation in humans
Source: Diabetes Obes Metab. 2015 Nov 20;18(1):72–81. doi: 10.1111/dom.12585 (PMC4710848; doi:10.1111/dom.12585)
Supplement: Supplementary file 3 — Figure S3. (A) Mean pulse rate (measured every 15 min) of all 11 subjects during exposure to the cooling vest (blue bar), vehicle infusion in a warm room (red bar) and glucagon infusion in a warm room (green bar). (B) Change in mean aterial pressure [estimated from diastolic (DBP) and systolic (SBP) measurements as DBP + 1/3(SBP − DBP)] between the start and end of the experimental exposures of cooling vest, vehicle and glucagon infusions. [file dom0018-0072-sd3.docx]

**Supplemental Figure S3: Mean pulse rate and mean arterial pressure (MAP) by intervention**

Figure S3a shows the mean pulse rate (measured every 15 minutes) of all 11 subjects during exposure to the cooling vest (blue bar), vehicle infusion in a warm room (red bar) and glucagon infusion in a warm room (green bar). S3b shows the change in MAP [estimated from diastolic (DBP) and systolic (SBP) measurements as DBP + 1/3(SBP-DBP)] between the start and end of the experimental exposures of cooling vest, vehicle and glucagon infusions. Results are expressed as means ± SEM , * p<0.05 and ***p<0.001 compared with vehicle.
