# Supplementary material for: Glucagon increases energy expenditure independently of brown adipose tissue activation in humans
Source: Diabetes Obes Metab. 2015 Nov 20;18(1):72–81. doi: 10.1111/dom.12585 (PMC4710848; doi:10.1111/dom.12585)
Supplement: Supplementary file 4 — Figure S4. Effects of cold exposure and glucagon infusion in a warm room on energy expenditure separated into brown adipose tissue (BAT)‐positive (n = 8) and BAT‐negative (n = 3) groups. [file dom0018-0072-sd4.docx]

**Supplemental Figure S4:** **Effects of cold exposure and glucagon infusion in a warm room on energy expenditure (EE) separated into BAT positive (n=8) and BAT negative (n=3) groups.**

Resting metabolic rate was measured with an indirect calorimeter at the start and end of each intervention and the mean change from baseline are shown.
