# Supplementary material for: Glucagon increases energy expenditure independently of brown adipose tissue activation in humans
Source: Diabetes Obes Metab. 2015 Nov 20;18(1):72–81. doi: 10.1111/dom.12585 (PMC4710848; doi:10.1111/dom.12585)
Supplement: Supplementary file 5 — Figure S5. Average deltoid (control) region of interest temperature (°C) (A–C) and neck (brown adipose tissue‐positive) region of interest temperature (°C) (D–F). [file dom0018-0072-sd5.docx]

**Supplemental Figure S5:** **Average deltoid (control) region of interest (ROI) temperature (^o^C) (S5a-c) and neck (BAT positive) ROI temperature (^o^C) (S5d-f).**

Results are shown for baseline (Run A, black bar) and end intervention (Run C, white bar) for each visit type. Each run represents a 10-minute thermal recording, with stills extracted every 30 seconds and an average reading of the upper 10% pixels calculated. Results are shown for n=8 BAT positive subjects (although there was no difference in the BAT positive versus BAT negative groups in the deltoid ROI, as expected for the control region which was chosen because it is known to be devoid of underlying BAT). Results are expressed as means ± SEM; ns not significant and ***p<0.001 compared with baseline.
