# Supplementary material for: Glucagon increases energy expenditure independently of brown adipose tissue activation in humans
Source: Diabetes Obes Metab. 2015 Nov 20;18(1):72–81. doi: 10.1111/dom.12585 (PMC4710848; doi:10.1111/dom.12585)
Supplement: Supplementary file 6 — Figure S6. Scatter plots for brown adipose tissue (BAT) activity and energy expenditure (EE) rise. (A) BAT metabolic activity [MR(gluc)] (µmol/kg/min) averaged across the cold PET/CT scan against the percentage rise in cold‐induced EE (as measured on the calorimetry day, under the same conditions of cold exposure). (B) BAT MR(gluc) against glucagon‐induced rise in EE. [file dom0018-0072-sd6.docx]

**Supplemental Figure S6: Scatter plots for BAT activity and EE rise.**

S6a: BAT metabolic activity [MRgluc] (µmol/kg/min) averaged across the cold PET/CT scan against the % rise in cold-induced EE (as measured on the calorimetry day, under the same conditions of cold exposure).

| Pearson r | 0.07295 |
| --- | --- |
| 95% confidence interval | -0.6661 to 0.7397 |
| P value (two-tailed) | 0.8637 |

S6b: BAT metabolic activity [MRgluc] against glucagon-induced rise in EE

| Pearson r | -0.8575 |
| --- | --- |
| 95% confidence interval | -0.9970 to 0.5892 |
| P value (two-tailed) | 0.1425 |
